# Differential Regulation of c-di-GMP Metabolic Enzymes by Environmental Signals Modulates Biofilm Formation in *Yersinia pestis*

**DOI:** 10.3389/fmicb.2016.00821

**Published:** 2016-06-03

**Authors:** Gai-Xian Ren, Sai Fan, Xiao-Peng Guo, Shiyun Chen, Yi-Cheng Sun

**Affiliations:** ^1^MOH Key Laboratory of Systems Biology of Pathogens, Institute of Pathogen Biology, Chinese Academy of Medical Sciences and Peking Union Medical CollegeBeijing, China; ^2^Institute of Nutrition and Food Hygiene, Beijing Centre for Disease Control and PreventionBeijing, China; ^3^Key Laboratory of Special Pathogens and Biosafety, Center for Emerging Infectious Diseases, Wuhan Institute of Virology, Chinese Academy of SciencesWuhan, China

**Keywords:** environmental signals, biofilm, c-di-GMP, diguanylate cyclases, phosphodiesterase, *Yersinia pestis*

## Abstract

Cyclic diguanylate (c-di-GMP) is essential for *Yersinia pestis* biofilm formation, which is important for flea-borne blockage-dependent plague transmission. Two diguanylate cyclases (DGCs), HmsT and HmsD and one phosphodiesterase (PDE), HmsP are responsible for the synthesis and degradation of c-di-GMP in *Y. pestis*. Here, we systematically analyzed the effect of various environmental signals on regulation of the biofilm phenotype, the c-di-GMP levels, and expression of HmsT, HmsD, and HmsP in *Y. pestis*. Biofilm formation was higher in the presence of non-lethal high concentration of CaCl_2_, MgCl_2_, CuSO_4_, sucrose, sodium dodecyl sulfate, or dithiothreitol, and was lower in the presence of FeCl_2_ or NaCl. In addition, we found that HmsD plays a major role in biofilm formation in acidic or redox environments. These environmental signals differentially regulated expression of HmsT, HmsP and HmsD, resulting in changes in the intracellular levels of c-di-GMP in *Y. pestis*. Our results suggest that bacteria can sense various environmental signals, and differentially regulate activity of DGCs and PDEs to coordinately regulate and adapt metabolism of c-di-GMP and biofilm formation to changing environments.

## Introduction

*Yersinia pestis*, the causative agent of plague, is a unique Gram-negative bacterium that adopts an arthropod-borne route of transmission. *Y. pestis* maintains its existence by a cycle involving two hosts: a mammal (usually a rodent) and an insect (a flea). In the flea host, the bacteria can form a biofilm in the proventriculus (a valve between the midgut and esophagus), which interferes with and can eventually block the ingestion of blood (Hinnebusch et al., 1996; Jarrett et al., 2004; Hinnebusch and Erickson, 2008). As a result of this blockage-induced starvation, fleas with complete or partial blockage will repeatedly try to feed on hosts, resulting in regurgitation of ingested blood and bacteria into the bite site to infect the mammal (Bacot and Martin, 1914; Bacot, 1915). *Y. pestis* biofilm formation requires the *hmsHFRS* operon, which is responsible for biosynthesis of a polymeric β-1, 6-*N*-acetyl-D-glucosamine-containing extracellular polysaccharide (EPS; Hinnebusch et al., 1996; Darby, 2008).

As in many other bacteria, production of EPS in *Y. pestis* is dependent on the intracellular levels of cyclic diguanylate (c-di-GMP; Sun et al., 2011; Bobrov et al., 2014). c-di-GMP, an intracellular second messenger molecule, controls biofilm formation, EPS production, motility, virulence, and many other cellular processes (Hengge, 2009; Romling, 2012; Romling et al., 2013). c-di-GMP is synthesized by diguanylate cyclases (DGCs) that contain a GGDEF domain, and is hydrolyzed by phosphodiesterases (PDEs) that contain an EAL or HD-GYP domain (Ryjenkov et al., 2005; Schmidt et al., 2005; Ryan et al., 2006). Bacteria are constantly exposed to changing environments, requiring that they coordinately regulate DGCs and PDEs levels to adjust the intracellular concentration of c-di-GMP to changes in the environment. Some environmental signals, such as temperature, light, oxygen, amino acids, growth medium and growth conditions, regulate c-di-GMP level and biofilm formation (Bernier et al., 2011; Malone et al., 2012; Koestler and Waters, 2013, 2014; Townsley and Yildiz, 2015). c-di-GMP signaling networks usually contain a large number of DGCs and PDEs that contribute to changes in the intracellular concentration of c-di-GMP. This redundancy suggests that the intracellular level of c-di-GMP reflects the cumulative activities of a complex composite of different DGCs and PDEs. Thus it is challenging to study how the concentration of c-di-GMP is modulated by environmental conditions.

Unlike other bacteria, the *Y. pestis* genome only contains three genes encoding enzymatically functional DGCs and PDEs (Bobrov et al., 2011; Sun et al., 2011). *hmsT* and *hmsD*, which encode DGCs, are responsible for synthesis of c-di-GMP, while *hmsP*, which encodes a PDE, is responsible for degradation of c-di-GMP (Kirillina et al., 2004; Bobrov et al., 2011; Sun et al., 2011).

*hmsD* is a member of the *hmsCDE* operon and is transcribed by the *hmsC* promoter (Sun et al., 2011). HmsC, a periplasmic protein, inhibits inner membrane protein HmsD DGC activity (Bobrov et al., 2014; Ren et al., 2014), while the outer membrane protein HmsE counteracts with HmsC to activate HmsD (Bobrov et al., 2014). HmsT plays a dominant role in biofilm formation *in vitro*, while HmsD plays a major role in producing proventricular-blocking biofilm during flea colonization (Sun et al., 2011). Hfq, a small RNA chaperone involved in regulation of HmsT and HmsP expression (Bellows et al., 2012), regulates biofilm formation in the flea and *in vitro* (Bellows et al., 2012; Rempe et al., 2012). Recently we found that 3′ untranslated region (3′ UTR) negatively regulates the *hmsT* expression in response to high temperature, allowing *Y. pestis* to adjust biofilm formation to the temperature of the host environment (Zhu et al., 2016). Although, only three enzymes are involved in maintaining c-di-GMP homeostasis in *Y. pestis*, the environment signals and the mechanisms by which HmsT, HmsD and HmsP, are regulated to control biofilm formation are not well-studied. The only known environmental signal that affects the expression of these enzymes in *Y. pestis* is temperature: HmsT is highly expressed at 26°C but not at 37°C (Perry et al., 2004; Zhu et al., 2016).

Little is known about environmental conditions in the digestive tract of the flea (Perry and Fetherston, 1997). In this study, we chose 12 different environmental conditions *in vitro* to simulate the environmental conditions in the gut of flea. We evaluated the effects of these environmental conditions *in vitro* on regulation of the biofilm phenotype, the c-di-GMP level, and the expression of HmsT, HmsCDE, and HmsP in *Y. pestis*. We found that changes in the environment affected the regulation of HmsT, HmsCDE, and HmsP, resulting in altered levels of intracellular c-di-GMP and differences in biofilm formation. Our findings may help to understand the regulatory mechanism of c-di-GMP signaling and biofilm formation in *Y. pestis* and in other bacteria.

## Results

### *Y. pestis* Biofilm Formation in Various Environments

**FIGURE 1:**
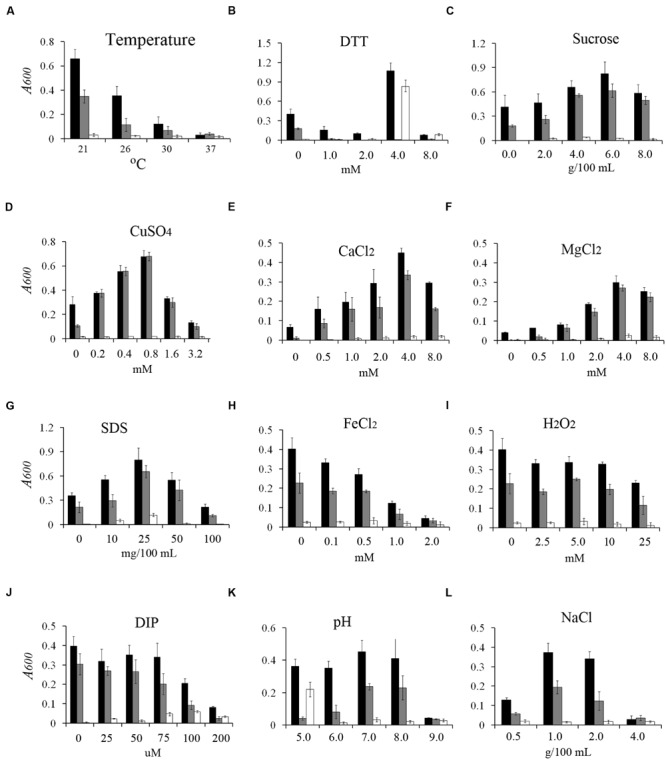
**Effect of environmental conditions on *Yersinia pestis* biofilm formation *in vitro*.** Relative amounts of adherent biofilm formed by the *Y. pestis*. KIM6+ parental strain (black bars) and its isogenic derivatives, the *hmsD* mutant (gray bars) and the *hmsT* mutant (white bars), in various environmental conditions, (**A**, bacteria culture at different temperature, **B–L**, media supplemented with different concentrations of DTT, sucrose, CuSO_4_, CaCl_2_, MgCl_2_, SDS, FeCl_2_, DIP, pH, NaCl). The mean and standard deviation of three independent experiments are indicated.

Biofilm formation might be regulated by different environmental signals. These signals might include the changes in pH, redox state, envelope stress, metal ions, and osmolarity and as well as other parameters. To determine the effect of different environmental signals on biofilm formation, we investigated the effect of 12 different environmental conditions on the growth and biofilm formation of *Y. pestis* KIM6+ *in vitro* (**Figure 1** and Supplementary Figure S1). Consistent with previous findings, biofilm formation of *Y. pestis* KIM6+ decreased as the temperature increased (**Figure 1A**). Addition of a low concentration of dithiothreitol (DTT; 1 or 2 mM) did not affect growth but decreased biofilm formation, while addition of 4 mM DTT resulted in slow growth but increased biofilm formation (**Figure 1B**). Biofilm formation was increased by addition of moderate concentration of sucrose, CuSO4, CaCl_2_, MgCl_2_, or sodium dodecyl sulfate (SDS) were added in to the media (**Figures 1C–G**), while bacterial growth was not strongly affected by these compounds (Supplementary Figures S1C–G). Conversely biofilm formation (**Figures 1C–G**) and bacterial growth (Supplementary Figures S1C–G) were inhibited when high concentrations of these compounds were employed. Biofilm formation but not bacterial growth was inhibited by FeCl_2_ (**Figure 1H** and Supplementary Figure S1H). Addition of non-toxic concentrations of H_2_O_2_, or 2,2′-dipyridyl (DIP; an iron chelator) did not markedly affect *Y. pestis* KIM6+ biofilm formation (**Figures 1I,J** and Supplementary Figures S1I,J). A low pH strongly inhibited *Y. pestis* KIM6+ growth but did not markedly affect its biofilm formation, while a high pH strongly repressed biofilm formation and growth (**Figure 1K** and Supplementary Figure S1K). In addition, biofilm formation was inhibited by both low and high concentrations of NaCl (**Figure 1L**), while bacterial growth was only strongly inhibited by a high concentration of NaCl (Supplementary Figure S1L).

To identify the roles of HmsT and HmsD in different environmental conditions, we assessed the biofilm formation in the *hmsT* and *hmsD* mutants in various environments. Consistent with the previous findings that *hmsT* plays a major role in biofilm formation *in vitro* (Bobrov et al., 2011; Sun et al., 2011), biofilm formation in the *hmsT* mutant was undetectable under most of environmental conditions tested (**Figure 1**), except in acidic (pH 5) or redox (addition of 4 mM DTT) environments (**Figures 1B,K**). On the other hand, biofilm formation was lower in the *hmsD* mutant in acidic (pH 5) and redox (addition of 4 mM DTT) environments (**Figures 1B,K**). These results suggest that *hmsD* might be upregulated in acidic or redox environments, and that it might be important for biofilm formation in these two environments. Addition of SDS, a membrane-disrupting detergent, not only markedly increased biofilm formation in the wild-type and *hmsD* mutant, but also resulted in detectable biofilm formation by the *hmsT* mutant (in the presence of 25 mg/100 mL SDS), indicating that SDS might not affect biofilm formation via the regulation of *hmsT* or *hmsD*, but via the regulation of *hmsP* or other unknown biofilm related genes (**Figure 1G**). Taken together, these results suggest that differential expression of *hmsT, hmsP*, and *hmsD* expression might occur in response to various environmental signals and that these three genes might function together to regulate biofilm formation.

### The Intracellular c-di-GMP Levels of *Y. pestis* at Various Environments

**FIGURE 2:**
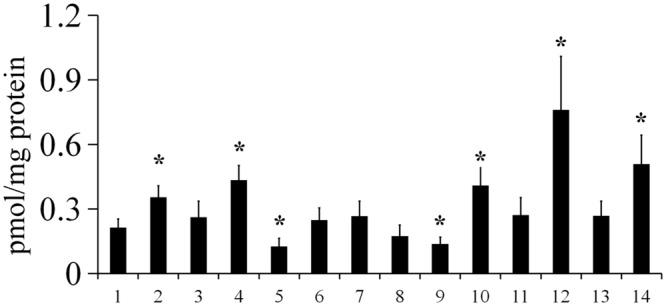
**The intracellular c-di-GMP level in *Y. pestis* in various environmental conditions.** c-di-GMP was extracted from *Y*. *pestis* grown in LB medium at 26°C (1), 21°C (7), or 37°C (8), or in LB medium supplemented with CaCl_2_ (2), MgCl_2_ (3), CuSO_4_ (4), FeCl_2_ (5), NaCl (9), sucrose (10), H_2_O_2_ (11), DTT (12), DIP (13), or SDS (14), or at pH 5 (6) at 26°C, and measured as described in the Experimental Procedures. ^∗^*P* < 0.05. The mean and standard deviation of three independent experiments are indicated.

Because biofilm formation in *Y. pestis* is affected by the c-di-GMP level (Kirillina et al., 2004; Simm et al., 2005; Bobrov et al., 2011; Sun et al., 2011), we reasoned that the effects of environmental conditions on biofilm formation might be due to changes in the cellular c-di-GMP level. Thus, we determined the intracellular c-di-GMP levels of *Y. pestis* under various environment conditions (4 mM CaCl_2_, 4 mM MgCl_2_, 2 mM FeCl_2_, 100 μM DIP, 1 mM CuSO4, 0.01% SDS, 6% sucrose, 4 mM DTT, 10 mM H_2_O_2_, or 4% NaCl, or modified to pH 5, and at high and low growth temperatures) that did not severely affect bacteria growth (Supplementary Figure S1). Consistent with the biofilm assay, the c-di-GMP level was increased by sucrose, CuSO_4_, CaCl_2_, SDS or DTT, decreased by FeCl_2_ or NaCl, and not affected by H_2_O_2_ or DIP (**Figure 2**). High concentration of FeCl_2_ might inhibited the function of Fur, the global ferric uptake regulator, that has been reported regulate *hmsT* expression, c-di-GMP level and biofilm formation in *Y. pestis* (Sun F. et al., 2012). Although, biofilm formation was increased by MgCl_2_ and decreased with temperature increase, the c-di-GMP levels were not significantly changed (**Figure 2**), suggesting that this environmental signal affects other unknown factors that regulate biofilm formation. It has been reported the *hmsHFRS* operon is downregulated by hyperosmotic stress (Han et al., 2005). Taken together these results suggested that many environmental signals affect the intracellular levels of c-di-GMP, which in turn, regulates biofilm formation in *Y. pestis*.

### Expression of PDE and DGCs at Different Growth Phase

**FIGURE 3:**
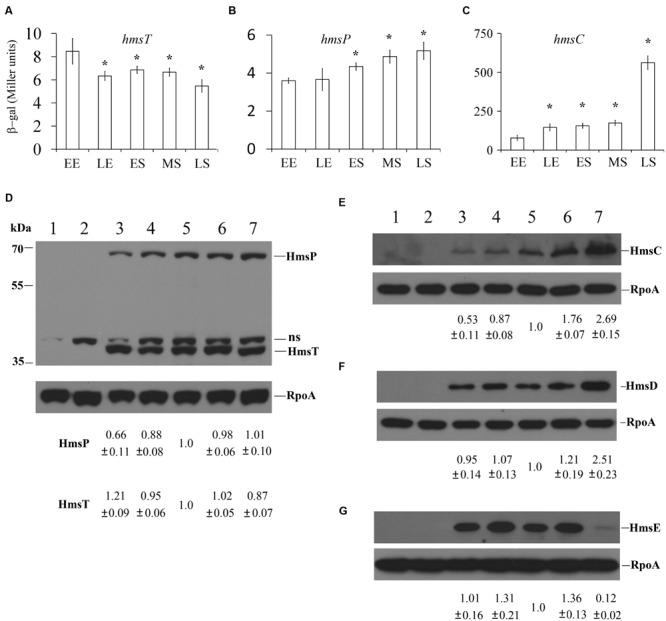
**Regulation of *hmsT, hmsP*, and *hmsCDE* according to the growth phase.** β-galactosidase activities of the *hmsT*::*lacZ* **(A)**, *hmsP*::*lacZ* **(B)**, and *hmsC*::*lacZ* **(C)** reporters in *Y*. *pestis*. EE, early phase; LE, late phase; ES, early stationary phase; MS, mid-stationary phase; and LS, late stationary phase. The protein levels of HmsT **(D)**, HmsP **(D)**, HmsC **(E)**, HmsD **(F)**, and HmsE **(G)** were detected by Western blotting. Lanes 1 and 2, *Y. pestis* KIM6+ in early exponential and mid-stationary phases, respectively; lanes 3–7, *Y. pestis* KIM6+ expressing HmsT-Flag **(D)**, HmsP-Flag **(D)**, HmsC-Flag **(E)**, HmsD-Myc **(F)**, and HmsE-HA **(G)** in early exponential (lane 3), late exponential (lane 4), early stationary (lane 5), mid-stationary (lane 6), and late stationary (lane 7) phases. The protein levels of HmsT, HmsP, and HmsCDE were quantitated using Image J and normalized according to the protein level of RpoA, the loading control. Numbers below the blots indicate the ratio of protein in the indicated sample with that in the sample collected at mid-stationary phase (Set 1.0) based on at least two independent experiments. ns, non-specific band.

Because DGCs and PDEs expression might be affected by growth phase, we determined *hmsT, hmsCDE*, and *hmsP* expression at the transcriptional and protein levels at different growth phases. To examine transcriptional expression of *hmsT, hmsCDE* and *hmsP*, we deleted the *Y. pestis lacZ* gene and constructed transcriptional fusions using *Escherichia coli lacZ* as the reporter as previously described (Sun Y.C. et al., 2012). All fusions were integrated into the chromosome at their native locus. Bacteria were grown in broth to early exponential (EE, OD_600_ 0.4–0.6), mid-exponential (ME, OD_600_ 1.0–1.2), early stationary (ES, OD_600_ 1.8–2.0), mid-stationary (MS, OD_600_ 2.4–2.6), and late stationary phases (LS, grown 14–16 h after mid-stationary phase), and β-galactosidase activity was assayed. Consistent with previous reports (Perry et al., 2004; Sun Y.C. et al., 2012), *hmsT::lacZ* transcription and *hmsP::lacZ* transcription were only slightly affected by growth phase (**Figures 3A,B**). *hmsT::lacZ* transcription was slightly lowered in the stationary phase (**Figure 3A**), while *hmsP::lacZ* transcription was slightly increased in the stationary phase (**Figure 3B**). *hmsC::lacZ* transcription was higher with bacterial growth and especially was significantly higher in the late stationary phase (**Figure 3C**).

Next, we examined HmsT, HmsC, HmsD, HmsE, and HmsP expression under different growth phases by performing Western blotting. 3 × Flag tags were added to the C-termini of HmsT, HmsP, and HmsC. 2 × Myc tags were added to the C-terminus of HmsD, and 3 × HA was added to the C-terminus of HmsE. Addition of these tags does not affect the function of these proteins (Ren et al., 2014; Guo et al., 2015 and data not shown). Each fusion protein was expressed under its own promoter on the chromosome at its native locus. HmsT-Flag and HmsP-Flag were constructed in the same strain; thus, the protein levels of HmsT and HmsP were detected at the same time using an anti-Flag antibody Supplementary Figure S2). Consistent with the results of *lacZ* reporter assay, the expressions of HmsT and HmsP were only slightly affected by growth phase, and HmsT expression was highest at early exponential phase, while HmsP expression was lowest at early exponential phase (**Figure 3D**). In addition, HmsC and HmsD were markedly increased in the late stationary phase (**Figures 3E,F**), which is consistent with the *lacZ* reporter assay (**Figure 3C**). However, HmsE expression was nearly undetectable in the late stationary phase (**Figure 3G**), suggesting that it is subject to post-transcriptional regulation. The transcriptional and protein levels of *hmsT, hmsP*, and *hmsCDE* were almost constant at the early stationary phase; therefore we collected bacteria in this phase and analyzed the expression of these genes in the following experiment.

### The Effects of Environmental Signals on Expression of HmsT, HmsP, and HmsCDE

**FIGURE 4:**
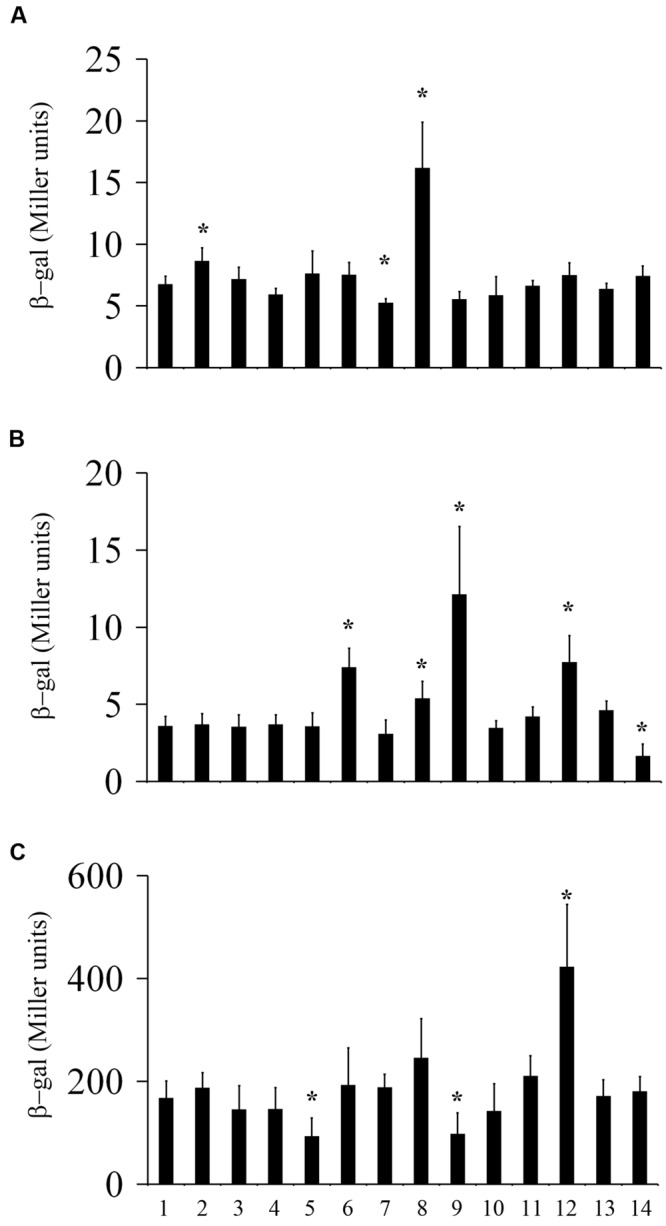
**Transcriptional regulation of *hmsT, hmsP*, and *hmsCDE* by environmental signals.** β-galactosidase activities of the *hmsT*::*lacZ* **(A)**, *hmsP*::*lacZ* **(B)**, and *hmsC*::*lacZ* **(C)** reporters in *Y*. *pestis* grown in LB medium at 26°C (1), 21°C (7), or 37°C (8), or in LB medium supplemented with CaCl_2_ (2), MgCl_2_ (3), CuSO_4_ (4), FeCl_2_(5), NaCl (9), sucrose (10), H_2_O_2_ (11), DTT (12), DIP (13), or SDS (14), or at pH 5 at 26°C. ^∗^*P* < 0.05. The mean and standard deviation of three independent experiments are indicated.

**FIGURE 5:**
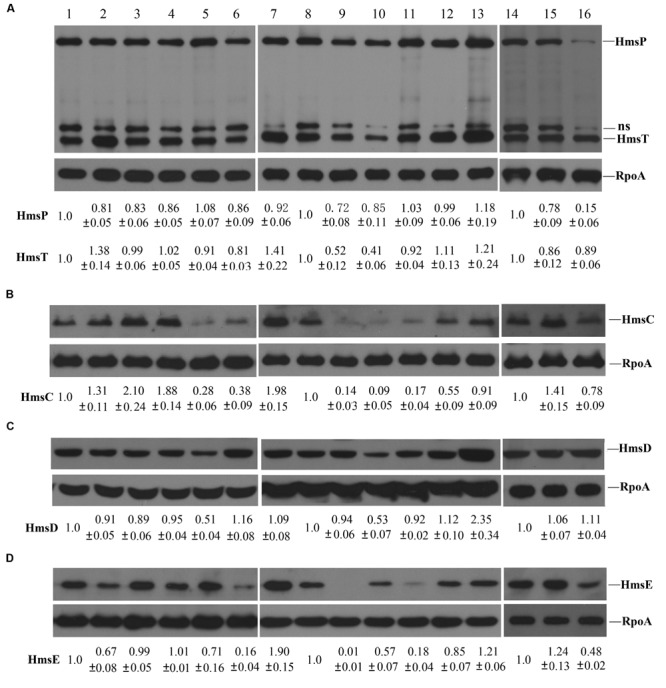
**Expression of *HmsT, HmsP*, and *HmsCDE* in various environmental conditions. (A)** The protein levels of HmsT, HmsP. **(B)** The protein levels of HmsC. **(C)** The protein levels of HmsD. **(D)** The protein levels of HmsE. The total protein lysates used for detection were prepared from cells grown in LB medium at 26°C (1, 8, and 14), 21°C (7), or 37°C (9), or in LB medium supplemented with CaCl_2_ (2), MgCl_2_ (3), CuSO_4_ (4), FeCl_2_ (5), NaCl (10), sucrose (11), H_2_O_2_ (12), DTT (13), DIP (15), or SDS (16), or at pH 5 at 26°C. The protein levels of HmsT, HmsP, and HmsCDE were quantitated using Image J and normalized according to the protein level of RpoA, the loading control. Numbers below the blots indicate the ratio of protein in the indicated sample with that in the sample collected from cells grown in LB medium at 26°C based on at least two independent experiments. ns, non-specific band.

*Yersinia pestis* biofilm formation and the intracellular c-di-GMP level were affected by environmental conditions. This might be because DGCs (HmsT and HmsD) and PDE (HmsP) are differentially regulated in different environments. To verify this, we first examined the effect of environmental conditions on HmsT expression. We monitored the transcriptional level of *hmsT* using the *lacZ* reporter and the HmsT protein level by performing Western blotting. *hmsT* transcription was increased by CaCl_2_, and a high temperature (37°C) but was decreased by a low temperature (21°C; **Figure 4A**). Consistent with the results of *lacZ* reporter assay, HmsT expression was not markedly affected (difference of less than 20%) by most of the environmental signals tested, but was 1.38-fold higher than in control cells after addition of CaCl_2_. The protein level of HmsT was increased at a low temperature (1.41-fold), and decreased at a high temperature (0.52-fold). This result is consistent with the previous reports that HmsT expression is post-transcriptionally regulated by temperature changes (Perry et al., 2004; Zhu et al., 2016). In addition, NaCl affected differentially the transcriptional levels and the protein levels of HmsT (**Figures 4A** and **5A**), suggesting that HmsT expression is also post-transcriptionally regulated by salinity stress.

Next, we examined the effect of environmental conditions on HmsP expression using the *lacZ* reporter and Western blotting. Transcription of *hmsP* was increased by a high concentration of NaCl, a high temperature (37°C), and an acid (pH 5) environment, but was decreased with addition of SDS (**Figure 4B**). Consistent with the *lacZ* reporter assay, HmsP expression was not markedly affected (difference of less than 30%) by most of the tested environmental signals (**Figure 5A**), but was decreased in the presence of SDS (0.15-fold). In addition, although transcription of *hmsP* was increased in the presence of a high concentration of NaCl, and in acidic and redox (addition of DTT) environments (**Figure 4B**), the protein levels of HmsP were not markedly affected in these environmental conditions (**Figure 5A**), suggesting that HmsP expression is post-transcriptionally regulated by these environments.

Finally, we determined the effects of environment conditions on HmsCDE expression. We first determined the promoter activity of *hmsC* using the *hmsC*::*lacZ* reporter. The promoter activity of *hmsC* was significantly decreased by high concentrations of FeCl_2_ or NaCl, but was increased by a redox environment (**Figure 4C**). We further detected the protein level of HmsD by performing Western blotting. Consistent with the results of *lacZ* reporter assay, the protein level of HmsD was not markedly affected (difference of less than 20%) by most of tested environmental signals (**Figure 5C**). Expression of HmsD was increased in the redox (2.35-fold) environments, and decreased in the high concentration of FeCl_2_ (0.51-fold) and NaCl (0.53-fold; **Figure 5C**), which is consistent with the *lacZ* reporter assay (**Figure 4C**). These results suggest that expression of HmsD is mainly regulated at the transcriptional level under these environmental conditions.

Because the activity of HmsD is regulated by HmsC and HmsE (Bobrov et al., 2014; Ren et al., 2014), we further determined the expression of HmsC and HmsE under various environmental conditions by performing Western blotting. By contrast to HmsD expression, which was not affected by most of the environmental conditions tested, the protein levels of HmsC and HmsE were markedly altered in the environmental conditions tested (**Figures 5B,D**). HmsC expression was increased by CaCl_2_ (1.31-fold), MgCl_2_ (2.1-fold), CuSO_4_ (1.88-fold), and DIP (1.41-fold), and by a low temperature (1.98-fold), but was decreased by FeCl_2_ (0.28-fold), NaCl (0.09-fold), and sucrose (0.17-fold), and at pH 5 (0.38-fold; **Figure 5B**). HmsE expression was markedly decreased by CaCl_2_ (0.67-fold), NaCl (0.57-fold), sucrose (0.18-fold), and SDS (0.48-fold) and at pH 5 (0.16-fold; **Figure 5D**). In addition, although neither the promoter activity of *hmsC* (**Figure 4C**) nor the HmsD protein level (**Figure 5C**) were significantly affected by the temperature, HmsC and HmsE expressions were markedly decreased by an increase in temperature (**Figures 5B,D**). These results suggest that the protein levels of HmsC and HmsE are more likely to be regulated than that of HmsD at the post-transcriptional level by environmental signals.

Taken together, these results suggest that HmsT, HmsP, and HmsCDE are differentially regulated by environmental signals, which might at least be partially responsible for changes in the intracellular c-di-GMP level and biofilm formation in *Y. pestis* in different environments.

### The Cumulative Effect of Environmental Signals on *Y. pestis* Biofilm Formation

**FIGURE 6:**
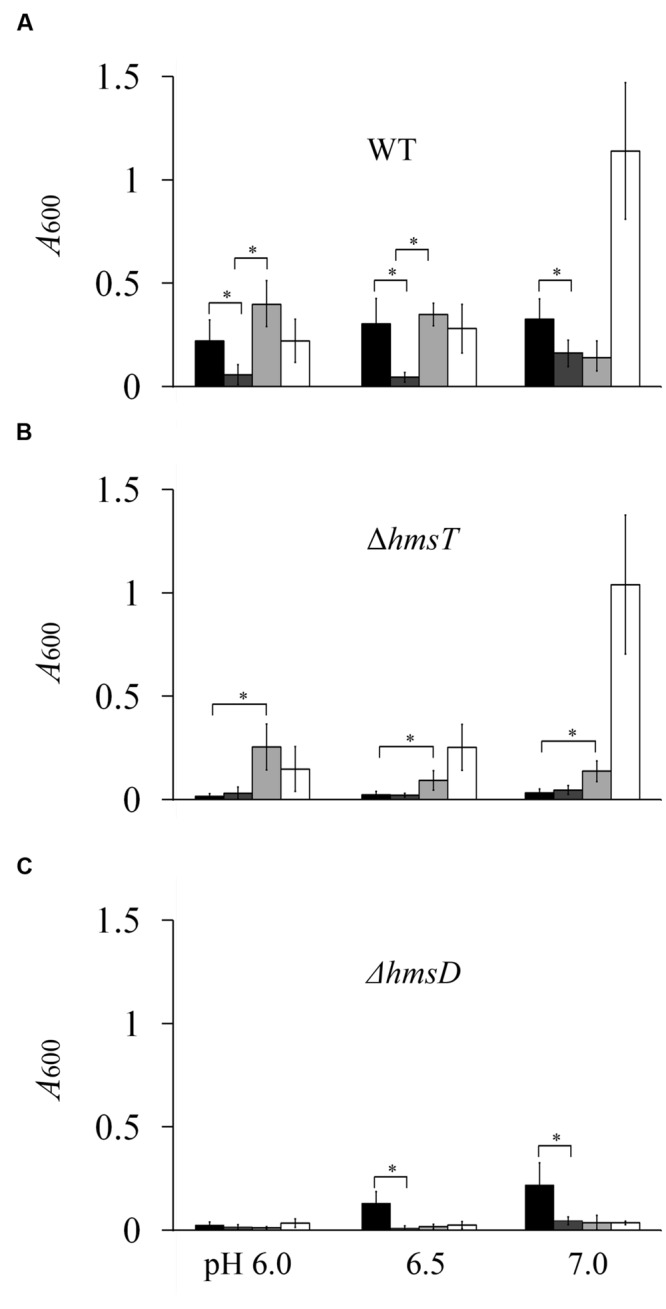
**The cumulative effect of environmental signals on *Y. pestis* biofilm formation.** Relative amounts of adherent biofilm formed by the *Y. pestis* KIM6+ parental strain **(A)** and its isogenic derivatives, the *hmsT* mutant **(B)** and the *hmsD* mutant **(C)**, in various environmental conditions. Black, dark gray, light gray, and white bars indicate cells were grown in medium supplemented with 0, 1, 2, and 4 mM DTT, respectively. The mean and standard deviation of three independent experiments are indicated. ^∗^*P* < 0.05

Biofilm formation can be affected by many environmental factors. *Y. pestis* forms a biofilm in the flea, however, little is known about the midgut physiology of the flea vector or environmental conditions in its digestive tract. The flea midgut pH is reportedly acidic (Perry and Fetherston, 1997), and probably between 6 and 7 (Zhou et al., 2012). Because HmsD plays a major role in the biofilm formation in acidic environment (**Figure 1K**), we believe that the acidic environment in the fleas might be one reason why *hmsD* plays a central role in these insects (Sun et al., 2011). A redox environment was another of the tested environmental conditions in which *hmsD* played a major role in *Y. pestis* biofilm formation; therefore we analyzed the effect of a combination of an acidic and redox environment on biofilm formation. Upon addition of 1 mM DTT, *Y. pestis* KIM6+ biofilm formation was decreased at various pHs (**Figure 6A**).

By contrast, addition of 2 mM DTT increased biofilm formation at pH 6.0, decreased formation at pH 7.0, and elicited nomarked change in formation at pH 6.5 (**Figure 6A**). Addition of 4 mM DTT strongly increased biofilm formation at pH 7.0, but did not significantly change biofilm formation at pH 6.0 or 6.5 (**Figure 6A**). In the presence of DTT, biofilm formation was almost undetectable in the *hmsD* mutant (**Figure 6C**). Biofilm formation in the *hmsT* mutant was almost undetectable in the absence of DTT or the presence of 1 mM DTT, but was significantly increased at pH 6–7 in the presence of 2 mM or 4 mM DTT (**Figure 6B**). In addition, biofilm formation in the *hmsT* mutant was decreased and increased by 2 and 4 mM DTT, respectively, as the pH increased from 6 to 7 (**Figure 6B**). Taken together these results suggest that various environmental signals can coordinately regulate HmsT, HmsP, and HmsD to modulate biofilm formation in *Y. pestis*.

Next, we analyzed the effects of combined environmental signals on HmsT, HmsP, and HmsCDE expression. HmsD expression was increased, whereas HmsC and HmsE expression was decreased by 2 mM DTT in an acidic environment (Supplementary Figure S3), which might account for the increased biofilm formation of the *hmsT* mutant at these environments. Although, biofilm formation in the *hmsD* mutant was decreased in the presence of 2 mM DTT at pH 6 and 6.5 (**Figure 6C**), the protein levels of HmsT and HmsP were not significantly affected (Supplementary Figure S3), suggesting that the enzyme activities of HmsT and HmsP might be regulated by these environmental signals. Taken together these results further suggested that HmsT, HmsP, and HmsD respond to various environmental signals and function together to regulate biofilm formation.

## Discussion

Bacteria in nature are exposed to a constantly changing environment and their ability to sense and react to these changes is important for their survival. A characteristic feature of c-di-GMP signaling networks is that a relatively large number of DGCs and PDEs contribute to changes in the intracellular c-di-GMP concentration. For example, *Vibrio cholera* encodes 53, *Pseudomonas aeruginosa* encodes 38, and *E. coli* encodes 29 putative DGCs or PDEs predicted to modulate c-di-GMP levels, respectively (Galperin et al., 2001). These DGCs and PDEs might be differentially regulated in response to changes in the environment. Thus, it is challenging to understand how the c-di-GMP level is controlled in these organisms. *Y. pestis* is an obligate pathogen that alternates between infection of mammals and fleas, and thus is only exposed to two different host environments. Thus, c-di-GMP signaling in *Y. pestis* is relatively simple and only three enzymes, two DGCs and one PDE, are involved in c-di-GMP metabolism. Thus, *Y. pestis* is a good model to study the mechanism by which bacteria sense changes in the environment to regulate c-di-GMP signaling and biofilm formation.

In this study, we systematically determined how environmental changes regulate c-di-GMP signaling and biofilm formation in *Y. pestis.* We tested 12 environmental signals and found that biofilm formation in *Y. pestis* was markedly affected by addition of non-toxic concentrations of CaCl_2_, sucrose, CuSO_4_, SDS, FeCl_2_, NaCl, and DTT and by temperature changes (**Figure 1**). The biofilm phenotype was consistent with the cellular c-di-GMP level (**Figure 2**), suggesting environmental signals regulate biofilm formation mainly through regulation of the c-di-GMP level. In addition, our results suggested that DGCs and PDEs differentially respond to environmental changes and that the intracellular level of c-di-GMP may reflect the cumulative activities of DGCs and PDEs. This is supported by the fact that addition of DTT and pH changes had different effects on biofilm formation in the wild-type, *hmsT* mutant and *hmsD* mutant (**Figures 1B,K** and **6**).

The cellular c-di-GMP level can be regulated by changes in the expression of c-di-GMP-metabolizing enzymes. The expression of genes encoding c-di-GMP metabolizing enzymes is affected by changes in environmental conditions. For example, transcription of genes encoding DGCs or PDEs in *E. coli* and *V. cholerae* decreases as the temperature increases (Sommerfeldt et al., 2009; Townsley and Yildiz, 2015). Here, we showed that the expression of HmsT, HmsP, and HmsD was differentially regulated by changes in environmental conditions at the transcriptional and post-transcriptional levels. Environmental changes changed the expression of HmsT and HmsP mainly at the post-transcriptional level. Although transcription of *hmsT* was increased by high temperature (**Figure 4A**), its protein level of HmsT was decreased (**Figure 5A**). We have reported that the 3′ UTR negatively regulates *hmsT* mRNA stability in response to temperature change. In addition, HmsT protein might be degraded by proteases at high temperature (Perry et al., 2004). The protein level of HmsT was increased by addition of DTT and decreased by addition of NaCl (**Figure 5A**), whereas the transcription of *hmsT* was unaffected by these environmental changes (**Figure 4A**). In addition, the transcription level of *hmsP* was increased by acidic (pH 5) and redox (addition of DTT) environments, by high temperature (37°C), and by a high concentration of NaCl (**Figure 4B**), but the protein level of HmsP was not markedly affected by these environmental changes (**Figure 5A**). The protein level of HmsD corresponded well with its transcriptional level (**Figures 4C** and **5C**), suggesting environmental changes regulate the expression of HmsD mainly at the transcriptional level. The expression the c-di-GMP genes is probably regulated at the transcriptional level, post-transcriptional level (mRNA degradation), translational level and post-transcriptional level (protein degradation), and together these regulations probably serve to controland adapt the levels of these enzymes and c-di-GMP level to changes in the environment.

The cellular c-di-GMP level can also be affected by changes in the activities of c-di-GMP-metabolizing enzymes. The activities of DGCs in *V. cholerae* are differentially regulated at low temperatures. It was suggested that the sensory input domains of these DGCs play an important role in the sensing low temperatures. The activity of HmsD is suggested to be reciprocally regulated by HmsC and HmsE. *hmsC, hmsD*, and *hmsE* are co-transcribed from the same *hmsC* promoter (Ren et al., 2014); however, their expression at the protein level differs depending on the environmental condition (**Figure 5**). The HmsD protein level was only moderately regulated by addition of DTT, FeCl_2_, or NaCl (**Figure 5C**), but HmsC and HmsE expression was moderately or strongly affected by most of environmental changes (**Figures 5B,D**). There are two possible explanations for this: (1) translation of HmsC and HmsE is particularly sensitive to environmental changes; and (2) more likely, the stabilities of HmsC, a periplasmic protein, and HmsE, an outer membrane protein, are particularly sensitive to environmental changes. Thus the c-di-GMP level and biofilm formation might quickly respond to the environmental changes through the regulation of HmsC and HmsE expression. For example, expression of HmsD, HmsT and HmsP was not markedly affected by changes in pH (**Figures 5A,C**), whereas expression of HmsC and HmsE was markedly decreased by an acidic environment (**Figures 5B,D**), which might account for the increased biofilm formation in the *hmsT* mutant in an acid environment. In addition, it has been reported that HmsE is important for the biofilm formation *in vivo* and *in vitro* (Bobrov et al., 2014), however, we found that HmsE does not play a significant role in the biofilm formation in *Y. pestis in vitro* (Ren et al., 2014). The medium that we used for the biofilm assay contained 4 mM CaCl_2_ and 4 mM MgCl_2_. A high concentration of CaCl_2_ or MgCl_2_ caused increased expression of HmsC but decreased expression of HmsE (**Figures 5B,D**), which might explain the different roles proposed for HmsE in previous reports.

HmsD plays a dominant role in *Y. pestis* biofilm formation in fleas, indicating that environmental conditions in the flea gut activate HmsD. Little is known about gut physiology and environmental conditions in the digestive tract of the flea. It has been suggested that the Mg^2+^ and Ca^2+^ levels in the flea midgut after consumption of blood meal are 0.8–1.0 mM and 2.0–2.5 mM, respectively (Rebeil et al., 2013). In addition, the pH in the flea midgut is reportedly probably acidic (Perry and Fetherston, 1997). Other basic parameters such as osmotic pressure and redox potential in the flea gut are unknown. The acidic environment might be one reason why *hmsD* plays a major role in controlling biofilm formation in the flea. This is supported by the fact that biofilm formation in *hmsT* and *hmsD* mutants was increased and decreased by an acidic environment, respectively (**Figure 1K**). There might be other unknown environmental signals that function together with an acidic environment to regulate the c-di-GMP signaling and biofilm formation in the flea gut. For example, a combination of an acidic environment and redox environment strongly activated HmsD-dependent biofilm formation but repressed HmsT dependent biofilm formation (**Figure 6**).

Using *Y. pestis* as a model strain, we showed that multiple environmental changes modulate c-di-GMP signaling and biofilm formation. The expression or activity of c-di-GMP-metabolizing enzymes in *Y. pestis* is differentially regulated in response to these environmental changes, resulting in alternation of the intracellular c-di-GMP level, which in turn modulates biofilm formation and allows *Y. pestis* to adapt to changing environments. This study may be helpful to understand the c-di-GMP signaling regulation in *Y. pestis* and other bacteria. The environmental conditions in the flea gut have not been fully investigated; thus, how *Y. pestis* regulates c-di-GMP signaling and biofilm formation to colonize fleas remains to be determined.

## Materials and Methods

### Bacterial Strains and Plasmids

The strains and plasmids used are shown in Supplementary Table S1. The *Y. pestis* KIM6+ (Deng et al., 2002), cured of the pCD1/pYV plasmid required for mammalian virulence, was used as the wild-type strain. *Y. pestis* mutants were made by a method to insert PCR products into the chromosome using the Red recombination system (Datsenko and Wanner, 2000; Sun et al., 2008). For strains containing reporter constructs *hmsC*::*lacZ, hmsT*::*lacZ* and *hmsP::lacZ*, were constructed on the chromosome using a modification of the Red method (Gerlach et al., 2007; Sun Y.C. et al., 2012). The primers used for construction of the above strains and plasmids are listed in Supplemental Table S2. All strains and plasmids were verified by PCR, DNA sequencing and plasmid complementation, as appropriate.

### *In Vitro* Biofilm Assays

Microtiter plate biofilm assays were performed as previously described with minor modifications (Ren et al., 2014). To determine the effects of Ca^2+^ and Mg^2+^, cells were grown in LB broth supplemented with appropriate concentration of MgCl_2_ or CaCl_2_ for 16–18 h at 26°C, and diluted to OD_600_ of 0.05 using the same media. To determine the effect of temperature, cells were grown in LB broth supplemented with 4 mM MgCl_2_ and 4 mM CaCl_2_ 16–18 h at various temperature, diluted to OD_600_ of 0.05 using the same media. To determine the roles of other environmental conditions, cells were grown in LB broth supplemented with appropriate concentration of 4 mM MgCl_2_ and 4 mM CaCl_2_ 16–18 h at 26, diluted to OD_600_ of 0.05 using the above media supplemented with appropriate concentrations of CuSO_4_, FeCl_2_, DIP, NaCl, sucrose, H_2_O_2_, SDS, or DTT, or modified to a different pH. Thereafter, 100 μL resuspended cells were aliquoted into 96-well polystyrene plates. The plates were incubated with shaking at 200 rpm for 24 h at 26°C or an appropriate temperature. OD_600_ was measured and then the plates were washed three times with distilled water. The adherent biofilm was stained with 0.01% crystal violet for 15 min. The dye was later re-dissolved with 80% ethanol and 20% acetone before the *A*_600_ was measured. Results from three independent experiments with at least three replicates per experiment were analyzed by a one-way analysis of variance (ANOVA) with Dunnett’s post-test.

### β-galactosidase Assays

β-galactosidase activities were measured as previously described (Sun Y.C. et al., 2012). Overnight cultures of *Y. pestis* harboring appropriate *lacZ* reporters were diluted with 50 mL of LB broth supplemented with 4 mM CaCl_2_, 4 mM MgCl_2_, 2 mM FeCl_2_, 100 μM DIP, 1 mM CuSO_4_, 0.01% SDS, 6% sucrose, 4 mM DTT, 10 mM H_2_O_2_, or 4% NaCl, or modified to pH 5, and grown at an appropriate temperature to the appropriate growth phase. ONPG (*o*-nitrophenyl-*b*-D-galactopyranoside) was cleaved by cell lysates at 37°C and this was in Miller units.

### Western Blotting

Western blotting was performed as previously described (Ren et al., 2014). Overnight cultures of *Y. pestis* were diluted 1:1000 in 50 mL LB broth supplemented with 4 mM CaCl_2_, 4 mM MgCl_2_, 2 mM FeCl_2_, 100 μM DIP, 1 mM CuSO4, 0.01% SDS, 6% sucrose, 4 mM DTT, 10 mM H_2_O_2_, or 4% NaCl, or modified to different pH 5 and grown at an appropriate temperature to the appropriate growth phase. Cells were collected and lysed by sonication. An appropriate amount of total proteins (100 ng total protein to detect HmsD and HmsE, 20 ng total protein to detect HmsC, HmsP, and HmsT) was loaded and separated on 10 or 15% SDS-PAGE gels, transferred to PVDF membrane (Millipore), analyzed by immunoblotting with antibodies against HA (Sigma), Flag (Invitrogen), Myc (Invitrogen) or antiserum against RpoA, and detected with Immobilon Western HRP Substrate (Millipore). Results were quantitated by densitometry using NIH ImageJ and normalized according to the loading amount of RpoA.

### Measurement of c-di-GMP Levels

Intracellular c-di-GMP levels in *Y. pestis* were detected as previously described (Ren et al., 2014). Overnight cultures of *Y. pestis* strains KIM6+ were diluted to an OD600 of 0.05 in LB broth supplemented with 4 mM CaCl_2_, 4 mM MgCl_2_, 2 mM FeCl_2_, 100 μm DIP, 1 mM CuSO4, 0.01% SDS, 6% sucrose, 4 mM DTT, 10 mM H_2_O_2_, or 4% NaCl, or modified to pH 5 and grown at an appropriate temperature to OD_600_ of 0.8. Cell pellets were collected and resuspended in 50 μL of extraction buffer (40% methanol and 40% acetonitrile prepared in 0.1 M formic acid) per 48 mg of wet cell weight. The slurries were incubated for 30 min at -20°C, and insoluble material was removed by centrifugation at 4°C. The supernatants were neutralized by adding 4 μL of 15% NH_4_HCO_3_ per 100 μL of sample. Ten microliters of each sample was analyzed using liquid chromatography tandem mass spectrometry. Synthetic c-di-GMP (BIOLOG Life Science Institute, Bremen, Germany) was used as a standard. Results from three independent experiments were analyzed using a one-way ANOVA with Dunnett’s test.

## Author Contributions

Experiment designation: G-XR, Y-CS; Experiment carry out: G-XR, SF, X-PG, SC; Manuscript writing: G-XR, Y-CS, SF; Manuscript review and modification: G-XR, SC, X-PG,Y-CS.

## Conflict of Interest Statement

The authors declare that the research was conducted in the absence of any commercial or financial relationships that could be construed as a potential conflict of interest.
