# Supplementary material for: Differential Regulation of c-di-GMP Metabolic Enzymes by Environmental Signals Modulates Biofilm Formation in Yersinia pestis
Source: Front Microbiol. 2016 Jun 3;7:821. doi: 10.3389/fmicb.2016.00821 (PMC4891359; doi:10.3389/fmicb.2016.00821)
Supplement: Supplementary file 1 [file Data_Sheet_1.DOC]

| Supplementary Table S1. Strains used in this study | | | |
| --- | --- | --- | --- |
| Strain | Genotype and/or description | Reference or source | |
| *Y. pestis* |  | |  |
| KIM6+  CDY362  CDY469  CDY497  SY091  SY1540  SY1562  SY1824  SY2010  SY2025  SY2046  SY2057  SY2263  SY2304 | wild type (pCD1-)  Δ*hmsP::Kan*  Δ*hmsD::Kan*  Δ*hmsT::Cm*  Δ*lacZ::Cm hmsP*::*lacZ*  Chromosomal *hmsC-*Flag_3_  Chromosomal *hmsD-*Myc_2_  Chromosomal *hmsT-*Flag_3_, *hmsP-*Flag_3_  Δ*hmsP::Kan* Δ*hmsT::Kan*  Δ*hmsP::Kan* Δ*hmsD::Kan*  Chromosomal *hmsE-*HA_3_  Δ*lacZ::Cm*  Δ*lacZ::Cm hmsC*::*lacZ*  Δ*lacZ::Cm hmsT*::*lacZ* | | (Deng et al., 2002 )  (Sun et al., 2011)  (Sun et al., 2011)  (Sun et al., 2011)  (Sun et al., 2012)  This study  (Guo et al., 2015)  This study  (Sun et al., 2011)  This study  This study  (Sun et al., 2012)  This study  (Sun et al., 2012) |
|  |  | |  |

**References**

Deng, W., Burland, V., Plunkett, G., 3rd, Boutin, A., Mayhew, G.F., Liss, P. et al. (2002) Genome sequence of *Yersinia pestis* KIM. *J Bacteriol* **184**: 4601-4611.

Guo, X.P., Ren, G.X., Zhu, H., Mao, X.J., and Sun, Y.C. (2015) Differential regulation of the *hmsCDE* operon in *Yersinia pestis* and *Yersinia pseudotuberculosis* by the Rcs phosphorelay system. *Sci Rep* **5**: 8412.

Sun, Y.C., Guo, X.P., Hinnebusch, B.J., and Darby, C. (2012) The *Yersinia pestis* Rcs phosphorelay inhibits biofilm formation by repressing transcription of the diguanylate cyclase gene hmsT. *J Bacteriol* **194**: 2020-2026.

Sun, Y.C., Koumoutsi, A., Jarrett, C., Lawrence, K., Gherardini, F.C., Darby, C., and Hinnebusch, B.J. (2011) Differential control of *Yersinia* *pestis* biofilm formation in vitro and in the flea vector by two c-di-GMP diguanylate cyclases. *PLoS One* **6**: e19267.

Supplementary Table S2. Oligonucleotides used in this study

| **Construction of *lacZ* deletion**  atgacgtcacaggaaaaggtaccactccaggtgcaactgagtcttgtgtaggctggagctgcttcg  ttacaccttgtattgccaacagatttggtactgataggtttcacgcatatgaatatcctccttag  **Construction of *hmsT::lacZ* fusion**  gatttattagtctactgacagcacgatattatgcagagtaaattgcccgtcgttttacaacgtcg  tcaaggggaagactgtacatttgataattcatctttagcaaattccgtgtaggctggagctgcttc  **Construction of *hmsP::lacZ* fusion**  atttgaagcccagtatttcagcagcgctcaccacgtaagttaatgaccaaaatcattcccgtcgttttacaacgtcg  tcttgctgacaagtgttttctaatatgtagggacttcacagccgtgtaggctggagctgcttc  **Construction of *hmsC::lacZ* fusion**  tccctttttagcccaacaggaagcgtaaaaatgactaccgctactcccgtcgttttacaacgtcg  ttacagccgcaaaccggtaaataaaggacgttaggacgcggtgatcgtgtaggctggagctgcttc  **Construction of *hmsP-*FLAG**  gattataaagatcatgacatcgactacaaggatgacgatgacaagtaactctttgtatggcggtgtcca  gtcgatgtcatgatctttataatcaccgtcatggtctttgtagtcacttacgtggtgagcgctgc  **Construction of *hmsT-*FLAG**  gattataaagatcatgacatcgactacaaggatgacgatgacaagtgattaactcactgaacatacggacgc  gtcgatgtcatgatctttataatcaccgtcatggtctttgtagtcaggggaagactgtacatttgataattc |
| --- |

All sequences are in 5’-3’ orientation

Supplemental Fig. 1.

Effect of environmental factors on *Y. pestis* growth *in vitro*. The *Y. pestis* KIM6+ parental strain (black bars) and its isogenic derivatives, the *hmsD* mutant (grey bars) and the *hmsT* mutant (white bars), were grown in various environmental conditions, OD_600_was measured before crystal staining was performed.
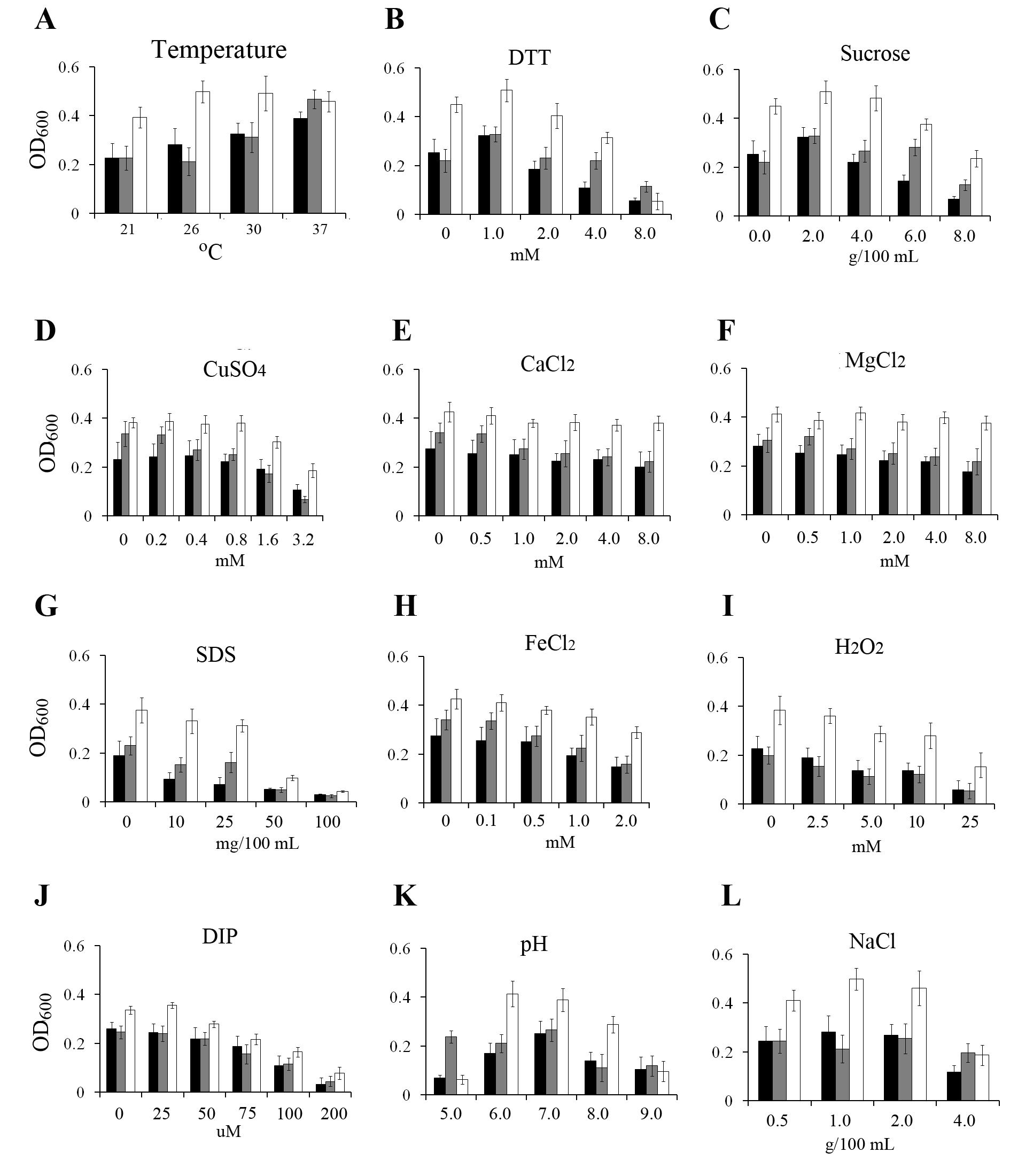


Supplemental Fig. 2.

Western blot analysis of HmsT and HmsP. The *Y. pestis* KIM6+ wild-type strain (1) and strains harboring HmsP-Flag (2), HmsT-Flag (3), and both fusion proteins (4) were analyzed by western blotting using an anti-Flag antibody. ns, non-specific band.


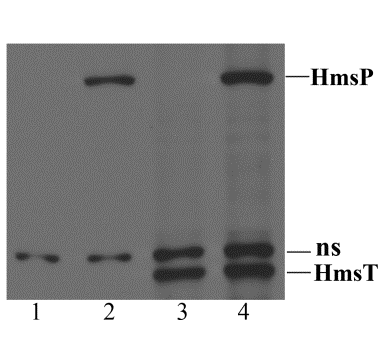


Supplemental Fig. 3.

The cumulative effect of environmental signals on expression of HmsT, HmsP and HmsCDE. The protein levels of HmsT (A), HmsP (A), HmsC (B), HmsD (C), and HmsE (E) were detected by western blotting. The total protein lysates used for detection were prepared from cells grown in LB medium at 26 °C (1), or in LB mediumat pH6.0, or pH6.5 in the presence of 2 mM DTT. The protein levels were quantitated using Image J and normalized according to the protein level of RpoA, the loading control. Numbers below the blots indicate the ratio of protein in the indicated sample with that in the sample collected from cells grown in LB medium at 26 °C based on at least two independent experiments. ns, non-specific band.


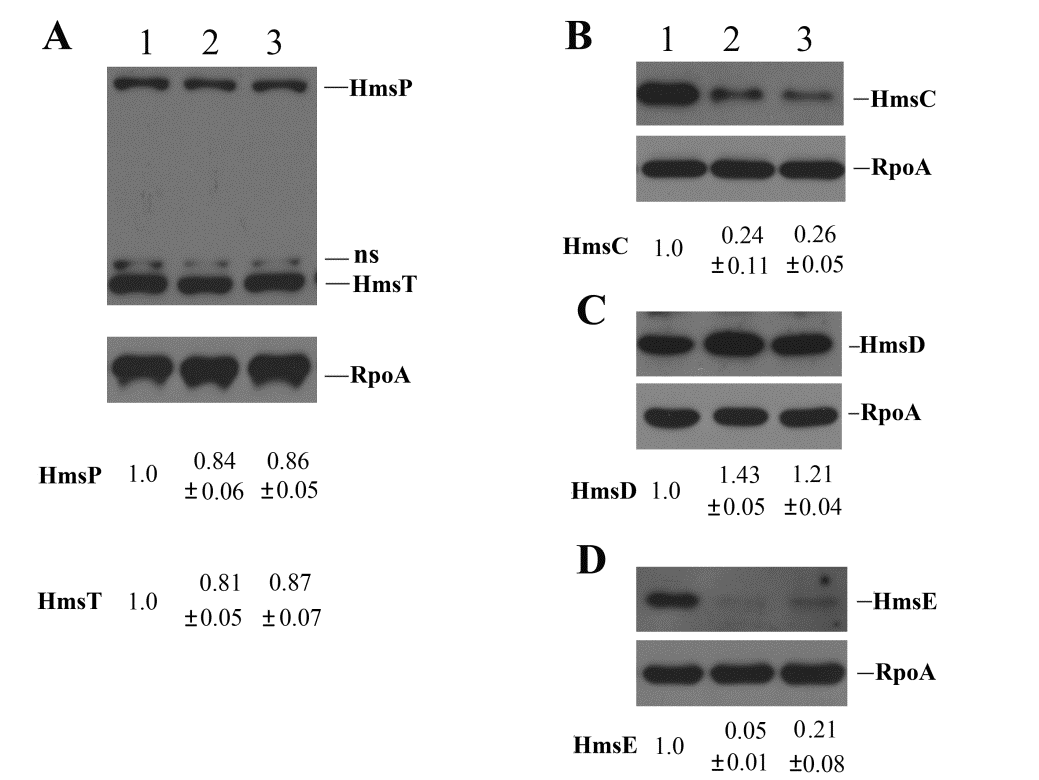


**Figure legends**

Supplemental Fig. 1.

Effect of environmental factors on *Y. pestis* growth *in vitro*. The *Y. pestis* KIM6+ parental strain (black bars) and its isogenic derivatives, the *hmsD* mutant (grey bars) and the *hmsT* mutant (white bars), were grown in various environmental conditions, OD_600_was measured before crystal staining was performed.

Supplemental Fig. 2.

Western blot analysis of HmsT and HmsP. The *Y. pestis* KIM6+ wild-type strain (1) and strains harboring HmsP-Flag (2), HmsT-Flag (3), and both fusion proteins (4) were analyzed by western blotting using an anti-Flag antibody. ns, non-specific band.

Supplemental Fig. 3.

The cumulative effect of environmental signals on expression of HmsT, HmsP and HmsCDE. The protein levels of HmsT (A), HmsP (A), HmsC (B), HmsD (C), and HmsE (E) were detected by western blotting. The total protein lysates used for detection were prepared from cells grown in LB medium at 26 °C (1), or in LB mediumat pH6.0, or pH6.5 in the presence of 2 mM DTT. The protein levels were quantitated using Image J and normalized according to the protein level of RpoA, the loading control. Numbers below the blots indicate the ratio of protein in the indicated sample with that in the sample collected from cells grown in LB medium at 26 °C based on at least two independent experiments. ns, non-specific band.
