# Supplementary figures and images for: Differential Regulation of c-di-GMP Metabolic Enzymes by Environmental Signals Modulates Biofilm Formation in Yersinia pestis
Source: Front Microbiol. 2016 Jun 3;7:821. doi: 10.3389/fmicb.2016.00821 (PMC4891359; doi:10.3389/fmicb.2016.00821)

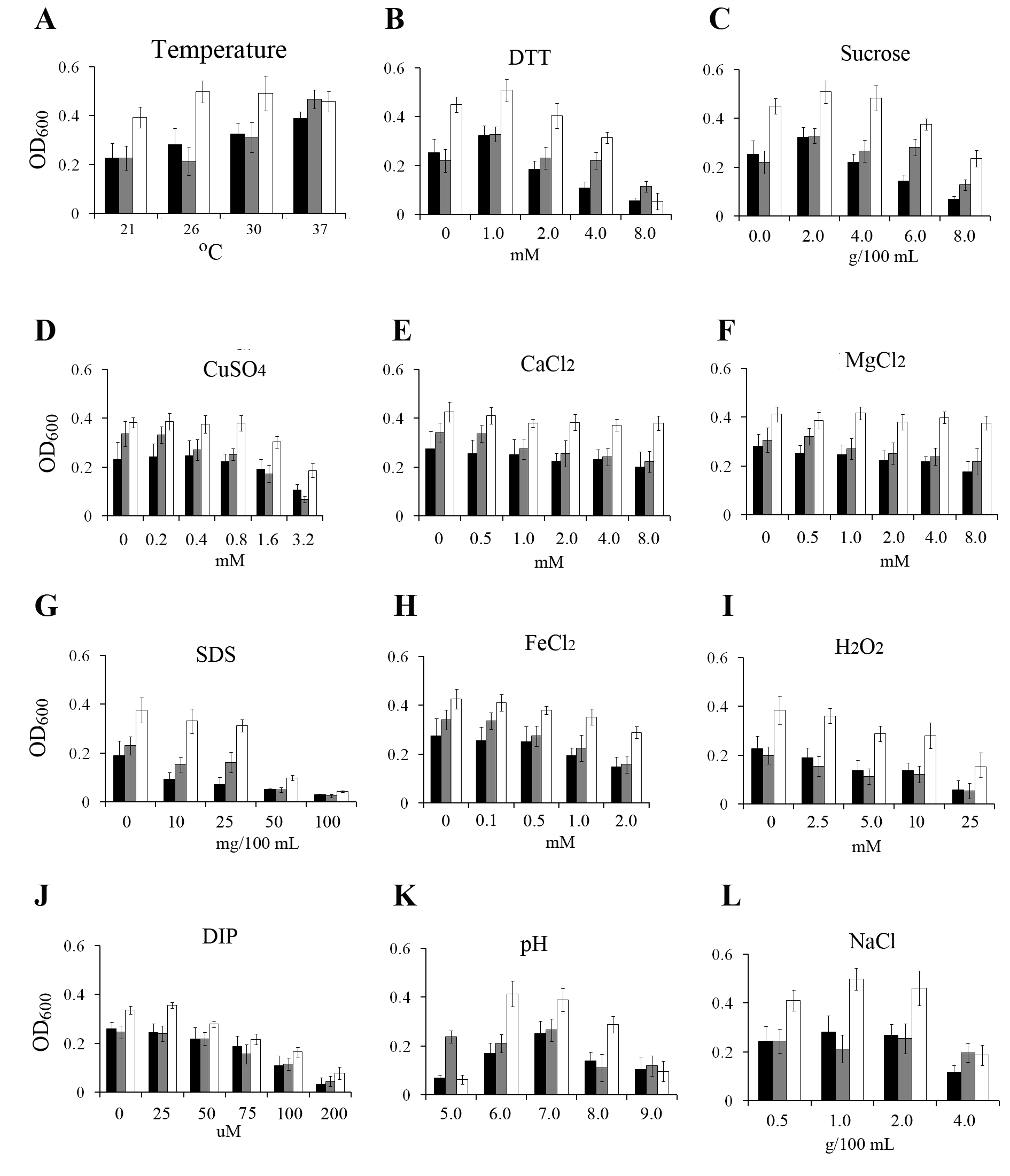

Supplement: Supplementary file 2 [file Image_1.TIF]

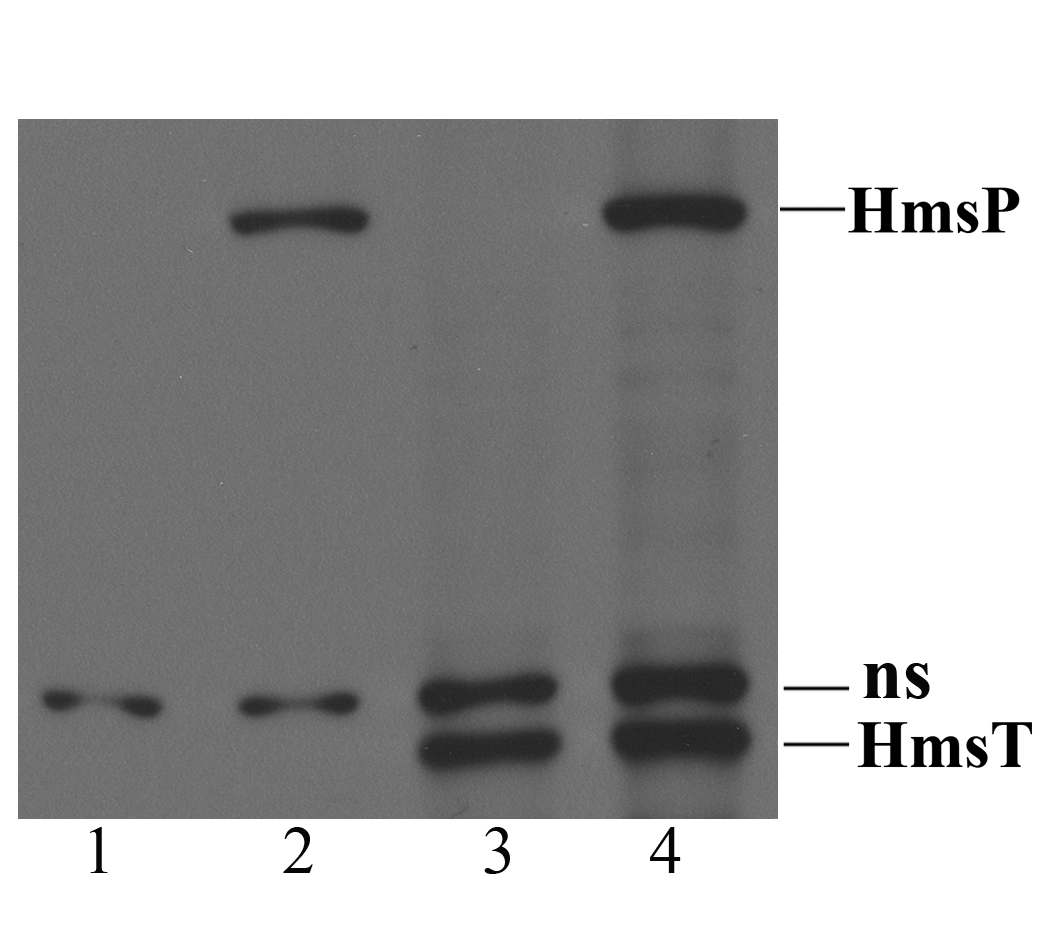

Supplement: Supplementary file 3 [file Image_2.TIF]

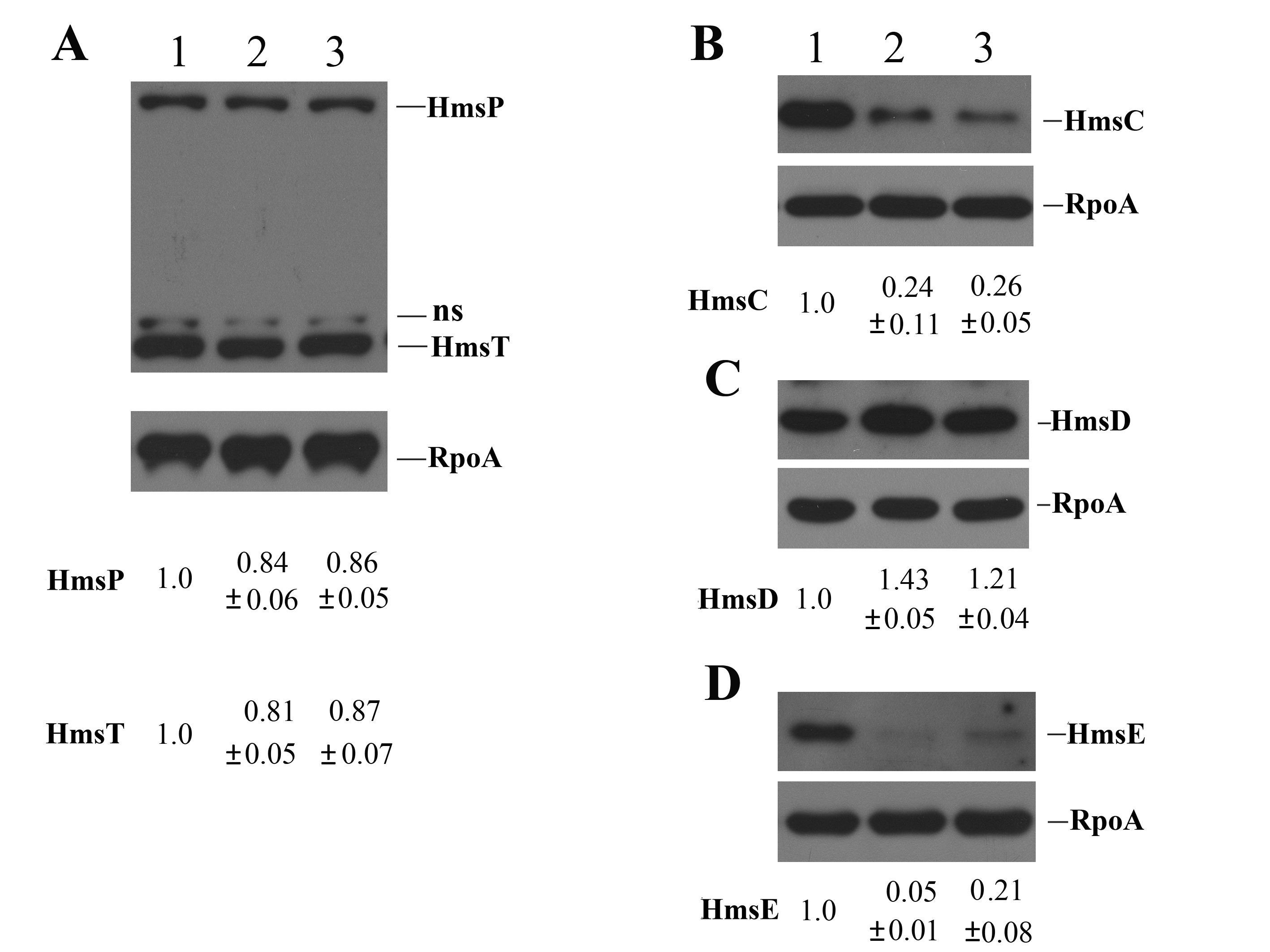

Supplement: Supplementary file 4 [file Image_3.TIF]
